# The impact of referring patients with drug-resistant focal epilepsy to an epilepsy center for presurgical diagnosis

**DOI:** 10.1186/s42466-023-00288-y

**Published:** 2023-12-14

**Authors:** Leonhard Mann, Felix Rosenow, Adam Strzelczyk, Elke Hattingen, Laurent M. Willems, Patrick N. Harter, Katharina Weber, Catrin Mann

**Affiliations:** 1 Epilepsy Center Rhine-Main, Center of Neurology and Neurosurgery, University Hospital Frankfurt, Goethe University, Frankfurt am Main, Germany; 2 Department of Neuroradiology, University Hospital Frankfurt, Goethe University Frankfurt, Schleusenweg 2-16, 60528 Frankfurt am Main, Germany; 3 LOEWE Center for Personalized Translational Epilepsy Research (CePTER), Goethe University, Frankfurt am Main, Germany; 4 Neurological Institute (Edinger Institute), University Hospital Frankfurt, Goethe University, Frankfurt am Main, Germany; 5 Centre for Neuropathology and Prion-Research, Ludwig-Maximilians-Universität München, München, Germany; 6 German Cancer Consortium (DKTK), Partner Site Frankfurt, German Cancer Research Center (DKFZ), Heidelberg, Germany; 7 Frankfurt Cancer Institute (FCI), Frankfurt am Main, Germany; 8 Center for Tumor Diseases, University Hospital Frankfurt, Goethe University, Frankfurt am Main, Germany

**Keywords:** Epilepsy, MRI, Epilepsy center, Non-expert MRI, Referral

## Abstract

**Background:**

Epilepsy surgery is an established treatment for drug-resistant focal epilepsy (DRFE) that results in seizure freedom in about 60% of patients. Correctly identifying an epileptogenic lesion in magnetic resonance imaging (MRI) is challenging but highly relevant since it improves the likelihood of being referred for presurgical diagnosis. The epileptogenic lesion’s etiology directly relates to the surgical intervention’s indication and outcome. Therefore, it is vital to correctly identify epileptogenic lesions and their etiology presurgically.

**Methods:**

We compared the final histopathological diagnoses of all patients with DRFE undergoing epilepsy surgery at our center between 2015 and 2021 with their MRI diagnoses before and after presurgical diagnosis at our epilepsy center, including MRI evaluations by expert epilepsy neuroradiologists. Additionally, we analyzed the outcome of different subgroups.

**Results:**

This study included 132 patients. The discordance between histopathology and MRI diagnoses significantly decreased from 61.3% for non-expert MRI evaluations (NEMRIs) to 22.1% for epilepsy center MRI evaluations (ECMRIs; *p* < 0.0001). The MRI-sensitivity improved significantly from 68.6% for NEMRIs to 97.7% for ECMRIs (*p* < 0.0001). Identifying focal cortical dysplasia (FCD) and amygdala dysplasia was the most challenging for both subgroups. 65.5% of patients with negative NEMRI were seizure-free 12 months postoperatively, no patient with negative ECMRI achieved seizure-freedom. The mean duration of epilepsy until surgical intervention was 13.6 years in patients with an initial negative NEMRI and 9.5 years in patients with a recognized lesion in NEMRI.

**Conclusions:**

This study provides evidence that for patients with DRFE—especially those with initial negative findings in a non-expert MRI—an early consultation at an epilepsy center, including an ECMRI, is important for identifying candidates for epilepsy surgery. NEMRI-negative findings preoperatively do not preclude seizure freedom postoperatively. Therefore, patients with DRFE that remain MRI-negative after initial NEMRI should be referred to an epilepsy center for presurgical evaluation. Nonreferral based on NEMRI negativity may harm such patients and delay surgical intervention. However, ECMRI-negative patients have a reduced chance of becoming seizure-free after epilepsy surgery. Further improvements in MRI technique and evaluation are needed and should be directed towards improving sensitivity for FCDs and amygdala dysplasias.

## Background

Epilepsy surgery is a widely accepted, long-established and evidence-based treatment option for patients with drug-resistant focal epilepsy (DRFE) that results in seizure freedom in ~ 60% of patients one year after epilepsy surgery [1]. In patients with epilepsy, resective surgery is associated with better seizure freedom rates than conservative treatment [2, 3]. Patients with epilepsy report an improved quality of life and social participation after surgical treatment [4]. Additionally, successful epilepsy surgery reduces mortality in this population [5, 6].

The outcome of surgical intervention is directly related to the epileptogenic lesion’s etiology. While surgical treatment of focal cortical dysplasias (FCD) type I leads to seizure freedom in only ~ 50% of patients, that of gangliogliomas (GGs) is associated with postoperative seizure freedom in ~ 80% of patients [1, 7]. Furthermore, a long duration between epilepsy onset and surgical intervention is associated with a worse postoperative seizure outcome. The chance of seizure freedom in low-grade epilepsy-associated tumors (LEAT), vascular malformations and FCDs decreases with long preoperative epilepsy duration [7, 8].

Identifying and etiologically classifying epileptogenic lesions in cerebral magnetic resonance imaging (cMRI) is challenging, especially for radiologists without specific epileptological expertise and if localizing information from other sources such as video electroencephalography (EEG) monitoring are lacking. Previous studies reported that non-expert radiologists failed to recognize epileptogenic lesions in > 60% of patients[9, 10]. Additionally, cMRI protocol selection appears to improve the sensitivity of cMRI, significantly reducing false cMRI-negative patients [9–11].

The unsuccessful identification of an epileptogenic lesion could discourage general neurologists from referring patients with DRFE to an epilepsy center for presurgical diagnosis. Misinterpreting an epileptogenic lesion could lead to an incorrect prognosis and postsurgical outcome estimation. Therefore, we compared the sensitivity and accuracy of non-expert cMRI evaluations with epilepsy center cMRI evaluations for different histologically-confirmed epileptogenic lesion etiologies in patients undergoing resective epilepsy surgery and explored the postsurgical outcomes of different subgroups.

## Methods

### Study group

**Fig. 1:**
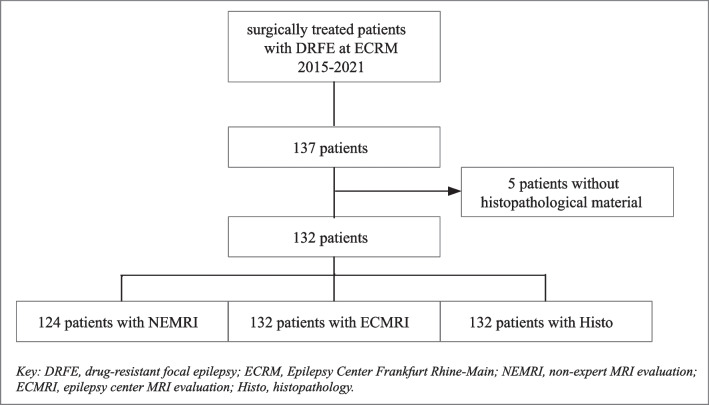
Flowchart of the patient selection and available data for the NEMRI, ECMRI, and histopathology modalities

This study included all patients with DRFE who underwent epilepsy surgery at the Epilepsy Center Frankfurt Rhine-Main (University Hospital Frankfurt, Germany) between 2015 and 2021. Five of these 137 patients were excluded because tissue could not be obtained for histopathologic workup due to stereotactic laser thermoablation therapy or histopathological analysis being not performed at our center. The resulting study cohort comprised 132 patients with complete presurgical workups and available histopathological findings at our center (Fig. 1).

### Non-expert MRI evaluation (NEMRI)

For each patient, the suspected cMRI diagnosis before consultation at our epilepsy center was collected. The cMRI diagnosis was based on imaging in an outpatient or non-specialized clinical setting with evaluation by radiologists without epilepsy-specific expertise (NEMRI). Data was gathered from written findings and clinical reports. This data was unavailable for eight patients due to missing information in clinical reports or because the first available imaging had been performed at our center. The remaining 124 patients had received NEMRI before consultation at our epilepsy center.

### Epilepsy-center MRI evaluation (ECMRI)

All patients underwent presurgical workup at our epilepsy center according to the epilepsy surgery guidelines as previously described [12] including cMRI evaluation by a neuroradiologist with epileptological experience based in our Neuroradiology Department. Presurgical workup used either new imaging at our center (axial T2, coronal T2 short tau inversion recovery [STIR], axial and coronal diffusion-weighted imaging [DWI], axial susceptibility-weighted imaging [SWI] in 3 mm, and 3D T2 fluid-attenuated inversion recovery [FLAIR] and T1 magnetization-prepared rapid acquisition with gradient echo [MPRAGE] in 1 mm in a 3 Tesla MRI; T1 post-contrast sequences were added if required) or reevaluation of original cMRI data if our neuroradiologists evaluated the external imaging to be of sufficient quality. Additional presurgical workup included at least five days of video-EEG-monitoring and neuropsychological evaluation for all patients. Fluorodeoxyglucose-positron emission tomography was obtained if considered helpful. Twenty-four patients underwent presurgical invasive long-term EEG monitoring using stereo-EEG or subdural grid electrodes.

Based on expert cMRI evaluations and this additional presurgical workup, the suspected diagnosis after consultation with our epilepsy center was obtained for each patient (ECMRI). MRIs were not reexamined for this study.

### Histopathology

Each patient’s final histopathological diagnosis was collected from written neuropathological reports. Histological and immunohistochemical processing included hematoxylin–eosin (HE), glial fibrillary acidic protein, cluster of differentiation (CD) 34, RNA binding fox-1 homolog 3 as well as neurofilament in non-neoplastic lesions and HE, isocitrate dehydrogenase 1, ATRX chromatin remodeler, microtubule-associated protein 2, oncohistone H3K27M, oncohistone H3K27 methyl group 3, CD34 as well as marker of proliferation Ki67 in neoplastic lesions. If required for diagnosis immunohistochemical stainings with monoclonal antibodies directed against CD68, myelin basic protein or allogaft inflammatory factor 1 were added.

Immunohistochemical studies were performed using a standard protocol on the immunostainer Discovery XT (Ventana Medical Systems, Oro Valley, AZ, USA) or Leica Bond III (Leica Biosystems, Nussloch, Germany) systems. No additional stainings were performed for this study.

### Additional clinical data

Additionally, sex, age, time from epilepsy onset to surgery, and postsurgical outcome at 12 months (according to the International League Against Epilepsy [ILAE] [13] and Engel [14] classifications) were collected for all patients. Follow-up data were available for 121 patients.

### Statistical analysis

The sensitivities and specificities of NEMRI and ECMRI were assessed by comparing them with histopathological diagnoses. Discordance between cMRI and histopathology and MRI-sensitivity were computed for NEMRI and ECMRI. Chi-square tests were used to detect statistically significant differences between NEMRI and ECMRI.

Both NEMRI and ECMRI diagnoses were divided into three groups: (1) MRI-negative findings, (2) discordant lesion etiology in MRI and histopathology, and (3) concordant MRI and histopathological lesion etiology. Postoperative outcomes at 12 months were compared between these three groups. Kruskal–Wallis tests were used to detect statistically significant differences between groups. Statistically testing was performed for group sizes > 5 individuals only. Additionally, the impact of MRI negativity on the time between disease onset and surgical intervention was examined by comparing epilepsy duration in MRI-negative patients with epilepsy duration in patients with recognized lesions in both NEMRI and ECMRI. Independent samples *t*-tests were used to detect statistically significant differences.

All results with *p* < 0.05 were considered statistically significant. All statistical analyses were performed in IBM SPSS Statistics 27 (IBM Corporation, Armonk, NY, USA). Sankey diagrams were created using the open-source freeware tool SankeyMATIC by Steve Bogart (www.sankeymatic.com).

## Results

### Study group

132 patients were included, of whom 63 were male and 69 were female. The mean patient age at surgery was 25.1 years (range 3 months–59 years). 23 patients were younger than 18 years at surgery of whom 9 were younger than 8 years old. Mean duration of epilepsy until surgery was 11.1 years (range 2 months–48 years).

At 12 months post-surgery, 66.9% of patients (*n* = 81) reported the absence of all seizures since surgery (seizure outcome Engel 1A/ILAE 1).

### NEMRI

NEMRI findings were available for 124 patients. No lesion was detected (MRI-negative) in 31.4% (*n* = 39). Suspected epileptogenic lesions were gliosis in 12.1% (*n* = 15), cavernoma in 11.3% (*n* = 14), unspecified tumor in 9.7% (*n* = 12), hippocampal sclerosis (HCS) in 8.1% (*n* = 10), dysembryoplastic neuroepithelial tumor (DNT) in 5.6% (*n* = 7), FCD in 4.9% (*n* = 6), unknown lesion in 4.0% (*n* = 5), and DNT or GG in 2.4% (*n* = 3). In addition, 5.6% (*n* = 7) were diagnosed with different tumor entities summarized as “other tumors” (two astrocytomas, one epidermoid, three unspecified gliomas, and one craniopharyngioma), and 4.8% (*n* = 6) showed different small entities summarized as “others” (two cysts, one hemimegalencephaly, one porencephaly, and one tuber).

### ECMRI

An ECMRI evaluation was available for all patients (*n* = 132). No lesion was detected (MRI-negative) in 2.3% (*n* = 3). Suspected epileptogenic lesions were FCD in 17.4% (*n* = 23), HCS in 16.7% (*n* = 22), gliosis in 12.1% (*n* = 16), cavernoma in 12.1% (*n* = 16), DNT in 7.6% (*n* = 10), GG in 6.8% (*n* = 9), finding consistent with amygdala dysplasia in 5.3% (*n* = 7), “DNT or GG” in 3.8% (*n* = 5), and encephalitis in 0.8% (*n* = 1). In addition, 8.3% (*n* = 11) showed different tumor entities other than LEATs summarized as “other tumors” (one astrocytoma, one epidermoid, one oligodendroglioma, one meningioma, one angiocentric glioma, and five unspecified low-grade gliomas), and 6.8% (*n* = 9) showed different small entities summarized as “others” (one cysts, one hemimegalencephaly, one porencephaly, one tuber, and five encephaloceles).

### Histopathology

Histological reports were available for all patients (*n* = 132). The final diagnosis was GG in 12.1% (*n* = 16), HCS in 12.1% (*n* = 16), DNT in 10.6% (*n* = 14), FCD type IIB in 9.1% (*n* = 12), gliosis in 8.3% (*n* = 11), cavernoma in 7.6% (*n* = 10), FCD IIA in 6.1% (*n* = 8), FCD IIIA in 3.0% (*n* = 4), finding consistent with amygdala dysplasia in 2.3% (*n* = 3), capillary telangiectasia in 1.5% (*n* = 2), unspecified FCD in 1.5% (*n* = 2), FCD IIIB in 0.8% (*n* = 1), and encephalitis in 0.8% (*n* = 1). In addition, 6.8% (*n* = 9) showed tumor entities other than LEATs summarized as “other tumors” (one anaplastic astrocytoma, one epidermoid, one oligodendroglioma, one meningioma, one angiocentric glioma, and four low-grade gliomas), and 3.0% (*n* = 4) showed different small entities summarized as “others” (one lissencephaly, one porencephaly, one encephalocele, and one tuber). No definite diagnosis could be determined for 14.4% (*n* = 19).

### Comparative analyses

#### Comparison of NEMRI and ECMRI with histopathological diagnoses

**Fig. 2:**
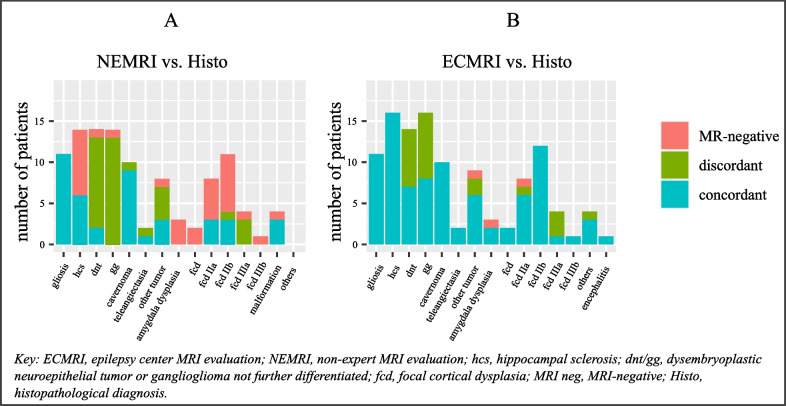
Distribution of concordant, discordant, and MRI-negative findings in NEMRI (**A**) and ECMRI (**B**) regarding different histopathological diagnoses

The most challenging diagnosis for NEMRI and ECMRI was determined by assessing their concordance with histopathological diagnosis. Only patients with unambiguous histopathological diagnoses were included in the comparative analyses (*n* = 113). Identifying amygdala dysplasias and FCDs was most challenging for both subgroups. NEMRIs missed 61.5% of FCDs (*n* = 16), and ECMRI missed 3.7% (*n* = 1). NEMRIs missed 57.1% (*n* = 8) of HCSs. However, ECMRIs identified all (100%, (*n* = 16) histopathologically confirmed HCSs (Fig. 2A+B).

**Fig. 3:**
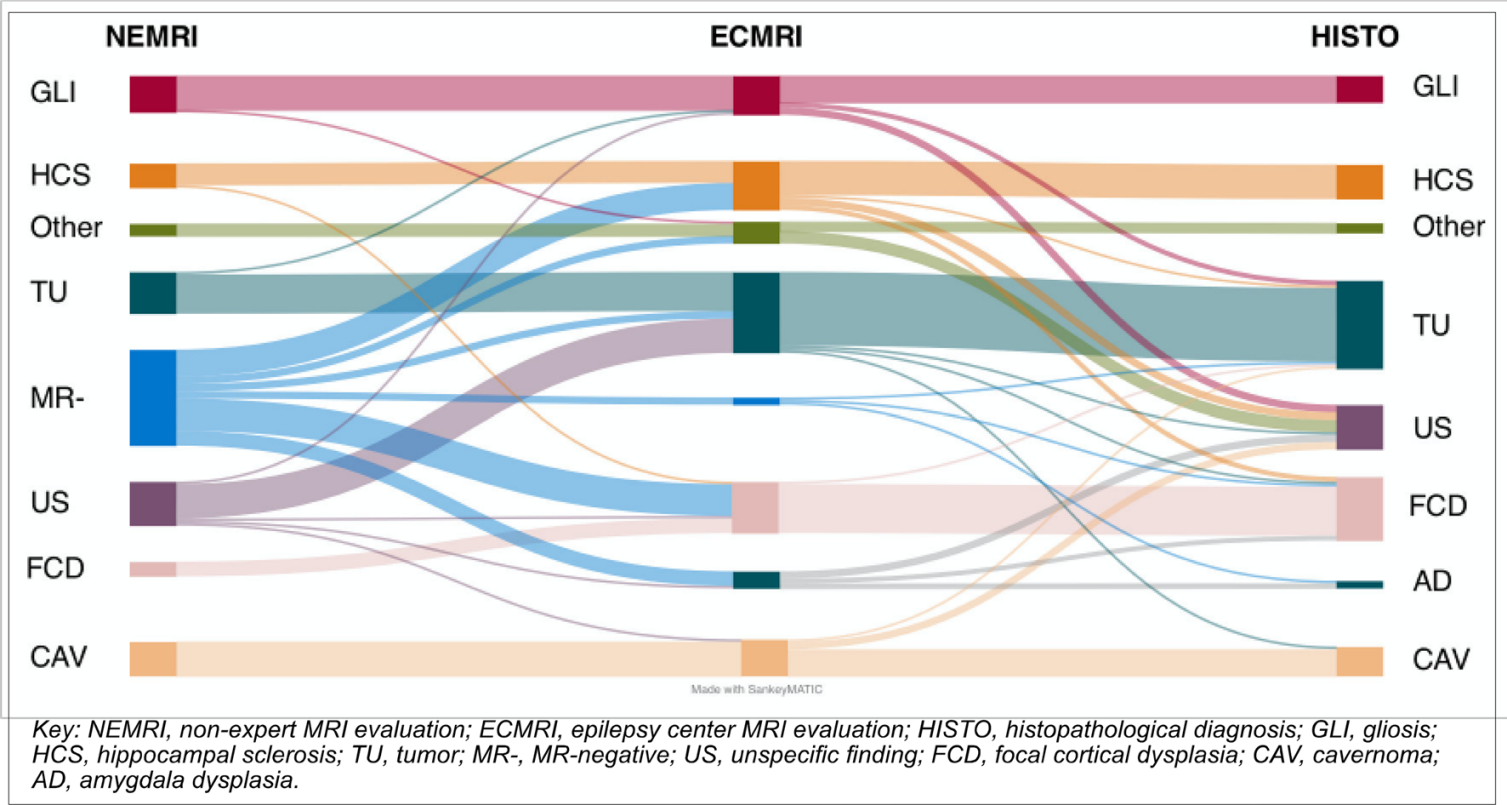
Sankey diagram visualizing the evolution of lesion etiologies from NEMRI to ECMRI and to final histopathological diagnosis

To better visualize the evolution of diagnoses from NEMRI to ECMRI and to final histopathological diagnoses, the etiologies were grouped into 8 groups. DNT, GG and other tumors were summarized as “tumor”. Unspecified tumors and unknown lesions were summarized as “unspecific”. Different small entities including encephalitis were summarized as “others”. NEMRI negative findings distributed the most. Gliosis and cavernoma remained in diagnosis of origin in most cases (Fig. 3).

#### Detailed analysis of histopathologically negative findings

For 19 patients no definite histopathological diagnosis could be determined. NEMRI-diagnosis of these patients was cavernoma in 15.8% (*n* = 3), gliosis in 15.8% (*n* = 3), unknown lesion in 10.5% (*n* = 2), and “others” in 10.5% (*n* = 2, both classified as cysts originally). 42.1% were NEMRI-negative (*n* = 8). For 5.3% (n = 1) no NEMRI diagnosis was available.

ECMRI diagnosis was “others” in 26.3% (*n* = 5, four classified as encephalocele and one as cyst originally), cavernoma, HCS, amygdala dysplasia and gliosis in 15.8% (*n* = 3) each, DNET vs. GG in 5.3% (*n* = 1) and FCD in 5.3% (*n* = 1).

Seizure freedom (Engel IA/ILAE 1) could be achieved in 50% of the subcohort (*n* = 8) 12 month postoperatively. Compared to patients with unambiguous histopathological diagnosis, who reached seizure freedom in 69.5% (*n* = 73) no significant difference could be shown (*p* = 0.071).

#### Comparison of NEMRI and ECMRI discordance and MRI-sensitivity

**Fig. 4:**
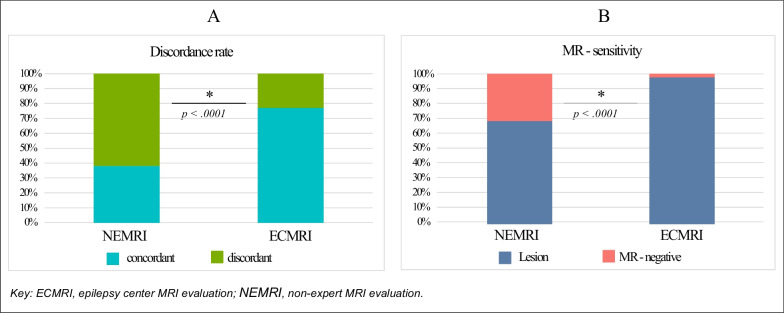
NEMRI and ECMRI discordance with histopathological diagnosis (**A**) and MRI-sensitivity (**B**)

NEMRI diagnoses and histopathological findings were discordant for 61.3% (*n* = 65). This proportion decreased to 22.1% (*n* = 25) after ECMRI evaluation. Therefore, ECMRI reduced the MRI-histopathology discordance rate by 39.2%. The Chi-square test indicated a significant difference (*p* < 0.0001; Fig. 4A).

NEMRIs identified an epileptogenic lesion in 68.6% (*n* = 85). This proportion increased to 97.7% (*n* = 129) after ECMRI evaluation. Therefore, ECMRI improved sensitivity by 29.1%. The Chi-square test indicated a significant difference between NEMRI- and ECMRI-sensitivity (*p* < 0.0001; Fig. 4B).

#### Comparison of NEMRI and ECMRI postsurgical seizure outcomes

**Fig. 5:**
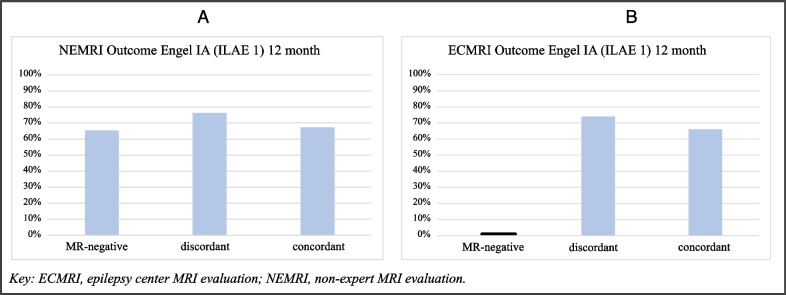
Outcomes of MRI-negative, discordant, and concordant to histopathology subgroups in NEMRI (**A**) and ECMRI (**B**)

Postoperative seizure outcomes at 12 months were compared between three groups: (1) MRI-negative findings, (2) discordant lesion etiology in MRI and histopathology, and (3) concordant lesion etiology in MRI and histopathology. No significant difference in outcomes among the three groups in NEMRI evaluation could be shown (*p* = 0.523; Fig. 5A). In contrast, outcomes after ECMRI evaluation differed between the three groups (Fig. 5B). While patients with discordant lesion etiology in ECMRI and histopathology showed seizure freedom in 73.9% (*n* = 17) and patients with concordant findings in 67.5% (n = 56), none of the patients with ECMRI-negative finding was seizure free 12 month postoperatively. No significant difference could be shown in outcomes among discordant and concordant lesion etiology (*p* = 0.580). Due to the small size of MR-negative subcohort no statistically testing was performed with the ECMRI-negative subcohort.

#### Epilepsy duration

**Fig. 6:**
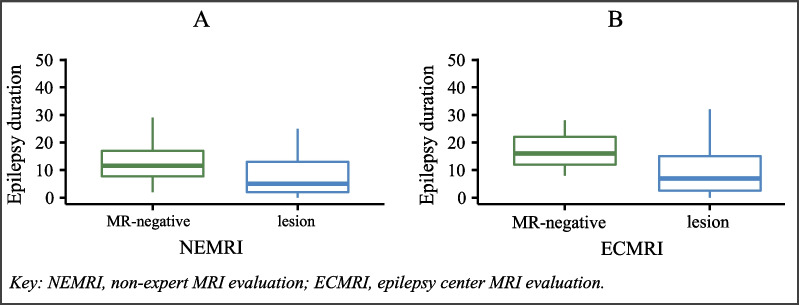
Impact of MRI-negative findings in NEMRI (**A**) and ECMRI (**B**) on epilepsy duration (in years)

Epilepsy duration from onset until surgical intervention was available for all patients (*n* = 132). In the entire cohort, the mean epilepsy duration was 11.1 years. The mean epilepsy duration was 13.6 years in NEMRI-negative and 17.3 years in ECMRI-negative patients. In contrast, in patients with recognized epileptogenic lesions, the mean epilepsy duration was 9.4 years for NEMRIs and 10.63 years for ECMRIs, not differing significantly between groups (NEMRI: *p* = 0.062, t-test; ECMRI: *p* = 0.280, t-test; Fig. 6A+B).

## Discussion

We showed that a substantial proportion of patients who underwent resective epilepsy surgery in our epilepsy center benefited from cMRI reevaluation by ECMRI, leading to a highly significant improvement of cMRI-sensitivity from 68.6 to 97.7%. The most common findings remaining unrecognized by NEMRI were FCD, amygdala dysplasia and HCS (Fig. 2A).

A crucial point in the epilepsy surgery process is the referral of patients from a general neurologist to an epilepsy center. In 2019, Steinbrenner et al. analyzed referral rates and reasons for non-referral in patients with DRFE [15]. Only 43% of eligible patients were recommended for presurgical evaluation by their treating neurologist at the epilepsy center of which 30% consented. The most frequent reasons for non-referral were a low seizure frequency and no expected success of the potential surgical intervention. About 50% of patients who rejected a referral to an epilepsy center had no epileptogenic lesion found in the non-expert MRI performed at a radiological practice outside an epilepsy center [15]. These data suggest a coherence between MRI-negative findings and non-referral to an epilepsy center. MRI-negative findings let physicians and patients doubt the success of epilepsy surgery, leading to a reluctance to referral to an epilepsy center for evaluation of surgical treatment options. Our data show that patients with findings classified as cMRI-negative by NEMRI do not have significantly worse seizure outcomes after epilepsy surgery than those with identified lesions in NEMRI (Fig. 5A). Therefore, patients found cMRI-negative by NEMRI should be referred for presurgical evaluation as early as cMRI-positive patients.

Additionally, a standardized epilepsy-adjusted cMRI protocol in an ambulant setting is important to exploit the full potential of imaging for epilepsy patients. NEMRI should be performed according to the MRI recommendations for epilepsy patients of the ILAE-neuroimaging task force: a standardized HARNESS protocol comprising millimetric 3D T1 and FLAIR weighted images and (sub-)millimetric 2D T2 high-resolution images [16]. Additionally SWI or T2* contrasts should be added to standard epilepsy imaging to improve sensitivity for small cavernomas typically associated with good surgical outcomes [7, 17, 18]. The practical implementation of the HARNESS protocol could be hindered by the medical compensation system, which does not compensate for sequences added to the smallest demanded amount of four sequences per cMRI in Germany. For epilepsy diagnosis with its special imaging requirements, compensation should be extended to at least 6 sequences to improve the sensitivity of ambulant MRI and accelerate diagnosis and best medical treatment.

To estimate the clinical value of the shown reduction in MRI-negative findings after referral to an epilepsy center, we compared outcomes between MRI-negative patients and patients with recognized lesion, including the subgroups with discordant and concordant MRI and histopathological findings. We found a difference in 12-month postoperative outcomes between ECMRI-negative patients and those with discordant or concordant MRI vs. histopathological findings (Fig. 5B). Restrictively, it must be mentioned that the outcome in our ECMRI-negative cohort does not go in hand with MR-negative outcomes described in literature, which could be explained by our small ECMRI-negative cohort (n = 3). Published postoperative seizure freedom rates in MRI-negative patients range from 40 to 50% [19–21] compared to 60–70% in patients with structural lesions on MRI [22]. According to this data, still patients with recognized lesion in ECMRI seem to have better outcomes than ECMRI-negative patients.

Interestingly, in our cohort, outcomes did not differ between patients with concordant and discordant ECMRI and histopathological findings (Fig. 5B). For interpretation of this observation, we have to consider that most discordant findings in ECMRIs in our cohort were GGs or DNTs. Both etiologies are assigned to LEATs, receive similar surgical intervention, and are associated with similarly good postoperative outcomes [8, 23]. Our results indicate, that with LEAT entities, the correct localization of epileptogenic lesions by EEG and MRI is much more important than the correct prediction of the LEAT subtype. Likewise, outcome of patients without definite histopathological diagnosis did not differ significantly from outcome with unambiguous histopathological finding. Conclusively, undefinite histological findings postoperatively does not necessarily result from missing the epileptogenic lesion intraoperatively. Inconclusive histopathologic diagnoses may result from difficulty in identifying appropriate specimens intraoperatively and sending them for further workup. A closer look at the preoperatively suspected diagnosis reveals a majority of rather subtle findings in ECMRI, that can appear unspecific in histopathology (amygdala dysplasia, encephalocele, gliosis). On the contrary, other suspected diagnosis in ECMRI not being confirmed by histopathological diagnosis (HCS, FCD) are well defined histopathological findings. In these cases a misinterpretation of MR-images must be considered.

Our results emphasize the importance of reducing MRI-negative findings in the presurgical workup. Therefore, we explored which diagnoses were most challenging for experts and non-experts. Identifying FCDs and amygdala dysplasias was the most challenging for both subgroups (Fig. 2A+B). NEMRI missed all histopathologically diagnosed amygdala dysplasias (*n* = 3), and ECMRI missed the lesion in one of three patients. FCDs were missed by NEMRI in 16 of 26 cases. In contrast, ECMRIs missed one of 27 FCDs.

Both FCDs and amygdala dysplasias are considered highly epileptogenic [24]. Epilepsy surgery leads to seizure freedom in ~ 60% of FCDs [7, 25, 26]. Seizure outcomes of amygdala dysplasias after epilepsy surgery have not been examined in detail until today, but mesiotemporal pathologies are associated with 60%–70% seizure freedom postoperatively [27, 28]. In our cohort, one of three patients with histopathologically confirmed amygdala dysplasia achieved Engel IA (ILAE 1) postoperatively. Therefore, improving diagnostic sensitivity for amygdala dysplasias and FCDs is vital to provide these patients best postoperative outome. Possible options for improving sensitivity for FCD are postprocessing, quantitative MRI, and ultra-high field (7 or 9 T) MRI, techniques that yet have to become accessible for clinical practice [29–35]. Additionally, it is crucial that epileptologists and radiologists exchange localizing information from EEG and semiology to improve MRI sensitivity to these subtle lesions.

Additionally, we observed an increased detection rate for HCS by ECMRI. While NEMRI missed 57.1% of HCS, ECMRI identified all (100%) histopathologically confirmed HCS (Fig. 2A+B). Besides higher expertise, which is hard to influence in the entirety of ambulant radiologists, the MRI protocol’s extent can influence MRI sensitivity [36]. High-resolution coronal T2-weighted images are highly sensitive for detecting HCS and are part of the epilepsy standard protocol at our epilepsy center. Following the recommendation of Bernasconi et al. [16], we suggest including coronal T2-weighted high-resolution images of temporal and mesiotemporal regions in standard epilepsy protocols to improve the detection of hippocampal pathologies.

Finally, we examined epilepsy duration in our cohort. Two comprehensive meta-analyses found a shorter disease duration as a predictive factor for better outcomes [7, 37]. Lamberink et al. highlighted that, unlike other known predictors of surgical outcome, epilepsy duration could be influenced by decisions made after the focal epilepsy diagnosis [7]. Regarding this aspect, we examined the impact of MRI-negativity on epilepsy duration at the time of referral to the epilepsy center. Patients with initial MRI-negative findings in NEMRI showed longer disease durations (three years longer on average) than patients with recognized lesions in NEMRI. When interpreting this result, we must remember that all our patients received ECMRI evaluations after referral, which revealed an unrecognized lesion in many patients that resulted in prompt surgery. We did not include NEMRI-negative patients, who never were referred to our epilepsy center, which would provide more realistic data. Looking at ECMRI evaluations, we found a mean delay of seven years in patients with MRI-negative findings (Fig. 6B). While the difference was again not statistically significant, seven years is relevant when suffering from a disease, especially considering the restrictions and risks associated with persistent epileptic seizures. Again, our results show the benefit of an early referral of patients with DRFE to an epilepsy center to reduce MRI-negative findings, shorten disease duration, and consecutively improve seizure outcomes.

This study had some limitations. Due to the differentiation of many histological subgroups, some only contained a few patients, limiting the power of our statistical analyses. Additionally, we only included patients with surgical treatment when examining the correlation between MRI and histological findings; we did not include patients with conservative treatment. Furthermore, all NEMRI findings were verified before surgery and adjusted by specialized neuroradiologists if needed. This approach especially biases our results on the outcomes of patients with NEMRI-negative and discordant findings.

## Conclusions

This study provides evidence that in patients with DRFE—especially those with initial MRI-negative findings—an early consultation with an epilepsy center, including an MRI evaluation by epilepsy expert neuroradiologists, is important to identify candidates for epilepsy surgery. Our data points out, that negative findings in NEMRI do not preclude seizure freedom postoperatively. Consequently, patients with DRFE that remain MRI-negative after initial NEMRI should be referred to an epilepsy center for presurgical diagnosis. Nonreferral based on NEMRI negativity may harm such patients and delay surgical intervention. However, ECMRI-negative patients have a reduced chance to become seizure-free after epilepsy surgery. Further improvements in MRI technique and evaluation are needed and should be directed towards improving sensitivity for FCD and amygdala dysplasia.

## Data Availability

The datasets used analyzed during the current study are available from the corresponding author on reasonable request.

## Abbreviations

CD: Cluster of differentiation
cMRI: Cerebral magnetic resonance imaging
DNT: Dysembryoplastic neuroepithelial tumor
DRFE: Drug-resistant focal epilepsy
DWI: Diffusion-weighted imaging
ECMRI: Epilepsy center MRI evaluations
EEG: Electroencephalogram
FCD: Focal cortical dysplasia
FLAIR: Fluid-attenuated inversion recovery
GG: Ganglioglioma
HCS: Hippocampal sclerosis
HE: Hematoxylin eosin
ILAE: International League Against Epilepsy
LEAT: Low-grade epilepsy-associated tumors
MPRAGE: Magnetization-prepared rapid acquisition with gradient echo
MRI: Magnetic resonance imaging
NEMRI: Non-expert MRI evaluation
STIR: Short tau inversion recovery
SWI: Susceptibility-weighted imaging
